# P-2198. Effect of IV Fluids on Diuretic Need in Pediatric and Adult Dengue Patients: A Network Meta Analysis and Systematic Review

**DOI:** 10.1093/ofid/ofaf695.2361

**Published:** 2026-01-11

**Authors:** Sophia Costa, Leticia R Campos, Gisella Carpi, Jose Luis Boene, Thiago Netto, Oscar Hernández Rios, Taniela M Bes

**Affiliations:** Jose Lucas Municipal Hospital, Belo Horizonte, Minas Gerais, Brazil; Universidade de Ribeirão Preto (UNAERP), Ribeirão Preto, Sao Paulo, Brazil; Hospital de Clínicas de Porto Alegre (HCPA), Porto Alegre, Rio Grande do Sul, Brazil; Eduardo Mondlane University, Maputo, Maputo, Mozambique; Evandro Chagas National Institute of Infectious Diseases (Fiocruz), Rio de Janeiro, Rio de Janeiro, Brazil; Ricardo Palma University, Lima, Lima, Peru; MetroWest Medical Center, Framighan, MA

## Abstract

**Background:**

Dengue fever (DF), transmitted by *Aedes aegypti* and *Aedes albopictus,* mostly in tropical and subtropical regions, causes high fever, muscle cramps, and joint pain. Fluid resuscitation is the mainstay of treatment, with Ringer’s Lactate (RL) being the first line recommended by the World Health Organization (WHO). Capillary leakage during resuscitation may happen and require diuretics, so the best IV fluid option for managing dengue remains in discussion.

**Figure 1:**
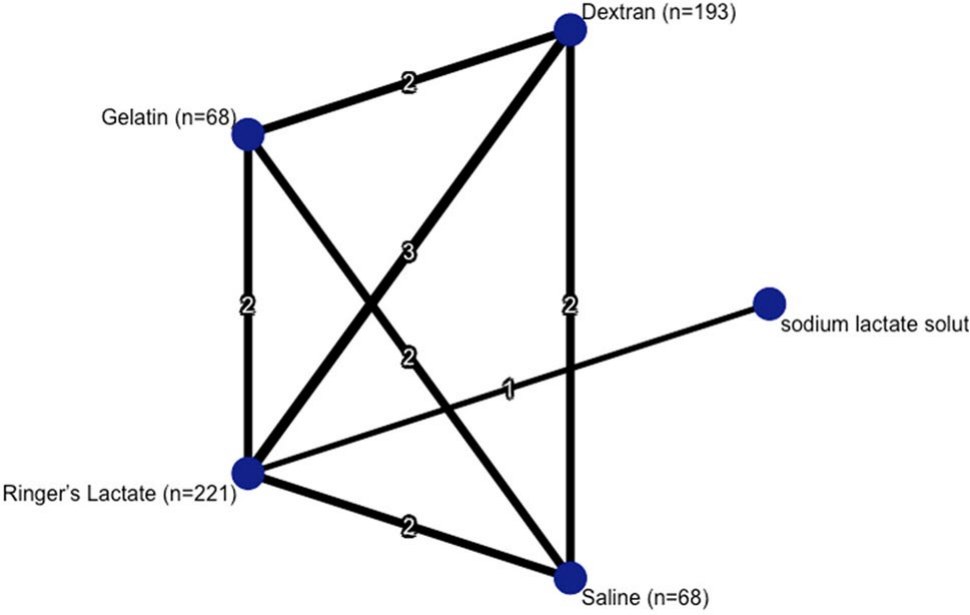
Network plor Network plot comparing the IV fluids and number of studies

**Figure 2:**
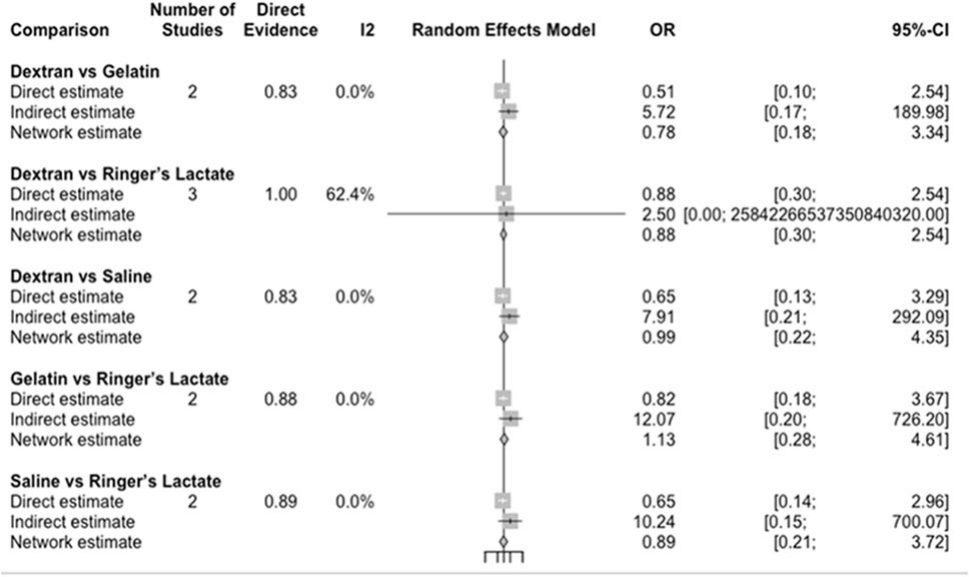
Forest Plot

Forest plot with indirect and direct effects on studies comparing IV fluids and the use of furosemide

**Methods:**

A literature search was performed in PubMed, Embase, and the Cochrane Library databases for Randomized Controlled Trials (RCTs) comparing RL with alternative IV fluids in patients with DF. The primary outcome was the use of furosemide after IV infusion. Data were analyzed using R, with heterogeneity assessed. Reference management and deduplication were done via PROSPERO, and risk of bias was evaluated using ROB-2.

**Results:**

Five RCTs were included, and direct/indirect comparisons were made (Figure 1). In this network meta-analysis of fluid therapies, Dextran vs Gelatin showed a non-significant trend favoring Dextran (Direct OR: 0.51; 95% CI: [0.10, 2.54]; Network OR: 0.78; [0.18, 3.34]). Dextran vs Ringer’s Lactate showed no clear difference (Direct and Network OR: 0.88), though the indirect estimate was highly imprecise. Dextran vs Saline also showed no meaningful difference (Network OR: 0.99; [0.22, 4.35]). Gelatin vs Ringer’s Lactate and Saline vs Ringer’s Lactate both showed no significant differences (Network ORs: 1.13 and 0.89, respectively), with wide confidence intervals. Heterogeneity was generally low (I² = 0%), except for Dextran vs Ringer’s Lactate (I² = 62.4%), indicating moderate inconsistency in that comparison (Figure 2).

**Conclusion:**

There was no significant association between the type of IV fluid administered and the need for furosemide due to plasma leakage. Further robust, direct comparisons are needed to strengthen the current evidence base

**Disclosures:**

All Authors: No reported disclosures

